# Shared genetic architecture between psychiatric and insulin-related traits in the general population

**DOI:** 10.1016/j.nsa.2026.107017

**Published:** 2026-06-17

**Authors:** Barbara Šakić, Izel Erdogan, Giuseppe Fanelli, Marieke Klein, Martina Arenella, Nina Roth Mota, Janita Bralten

**Affiliations:** a Department of Human Genetics, Radboud University Medical Center, Geert Grooteplein Zuid 10, Nijmegen, 6525 GA, the Netherlands; b Donders Institute for Brain, Cognition and Behaviour, Radboud University, Erasmusplein 1, Nijmegen, 6525 HT, the Netherlands; c Department of Medical Neuroscience, Radboud University Medical Center, Geert Grooteplein Zuid 10, Nijmegen, 6525 GA, the Netherlands; d Department of Medicine, University of Udine, Via Colugna, 50, Udine, 33100, Italy; e Department of Forensic and Neurodevelopmental Sciences, Institute of Psychiatry, Psychology and Neuroscience, King's College London, 16 De Crespigny Park, London, UK

**Keywords:** Insulin resistance, Psychiatric traits, Multivariate genetics

## Abstract

Psychiatric conditions, like Attention-Deficit/Hyperactivity Disorder (ADHD), Autism Spectrum Disorder (ASD), and Obsessive-Compulsive Disorder (OCD) are complex heritable disorders that frequently co-occur with insulin resistance (IR)-related conditions, such as obesity and type 2 diabetes mellitus. Traditional genetic case-control comparisons are challenged by the extent of heterogeneity and comorbidity within and across these conditions. In this study we used psychiatry-related traits in the general population in multivariate analyses to explore the potential genetic links with insulin-related traits.

Using large-scale genetic studies (N = 17,666–697,734) genomic structural equation modeling was applied to identify the factor structure best representing the joint genetic architecture of traits in the population that are linked to ADHD, ASD, and OCD, and five IR-related measures: body mass index (BMI), fasting plasma glucose (FPG), fasting plasma insulin, glycated haemoglobin (HbA1c), and the Homeostatic Model assessment for IR (HOMA-IR). Next, multivariate genome-wide association analyses were performed on the psychiatry-IR related factors to explore genetic associations, followed by functional mapping and annotation of the genetic results.

Factor analyses indicated that a three-factor model fitted the data best (χ2(df = 9) = 18.79, AIC = 56.8, CFI = 0.99, SRMR = 0.068). Two of the three genetic factors included both psychiatric and insulin-related traits, one factor connected ADHD symptoms with three IR-related measures (BMI, FPG, HbA1c) and another factor OCD symptoms with HbA1c. Genetic analyses revealed 57 genes significantly associated with the ADHD-IR factor (p < 2.961e-06) and one gene, *MTNR1B* (p = 3.44e-07), with the OCD/OCS-IR factor. Neurodevelopmental pathways were associated to the psychiatric and insulin-related factors and expression of the associated variants was enriched in brain tissues and during brain development.

These findings suggest a shared genetic liability between psychiatric symptoms and IR-related measures in the general population, indicating that the link between insulin resistance and psychiatry is not solely driven by disorder status and offering new perspectives on the underlying molecular genetics between them.

## Highlights

• Psychiatric conditions frequently co-occur with somatic conditions linked to insulin resistance (IR), like obesity and type 2 diabetes.
• Extensive genetic links exist between psychiatric conditions and IR-related conditions, but are challenged by heterogeneity and co-morbidity.
• We modeled shared genetics between psychiatric-like traits and IR-related measures using large-scale population-based data.
• We find shared genetics between psychiatric traits and IR-related traits demonstrating that the link is not solely driven by disorder status.
• The genetic findings point to genes relevant for neurodevelopment and expressed in the brain.

## Abbreviations

ADHD: Attention-Deficit/Hyperactivity Disorder
ADHD_total_symp: ADHD total symptom score
AIC: Akaike Information Criterion
ASD: Autism Spectrum Disorder
BA9: Brodmann Area 9
Beta: Beta value
BMI: Body Mass Index
CADM2: Cell Adhesion Molecule 2
CBCL: Child Behavior Checklist
CFA: Confirmatory Factor Analysis
CFI: Comparative Fit Index
CPNE4: Copine 4
df: Degrees of Freedom
EAGLE: EArly Genetics and Life course Epidemiology
EFA: Exploratory Factor Analysis
eQTL: expression Quantitative Trait Loci
FPG: Fasting Glucose
FPI: Fasting Insulin
FUMA: Functional Mapping and Annotation
GO: Gene Ontology
GWAS: Genome-Wide Association Study
HbA1c: Glycated haemoglobin
HOMA-B: Homeostatic model assessment of β-cell function
HOMA-IR: Homeostatic model assessment for insulin resistance
INFO: Info score
IR: Insulin Resistance
LD: Linkage Disequilibrium
MAF: Minor Allele Frequency
MAGMA: Multi-marker Analysis of GenoMic Annotation
MTNR1B: Melatonin Receptor 1 B
mTOR: Mechanistic target of Rapamycin
N: Sample size
N_eff_: : Effective sample size
N genes: Number of genes
NRXN1: Neurexin-1
OCD: Obsessive-Compulsive Disorder
OCS: Obsessive-Compulsive Symptoms
OCS_OCD: Obsessive-compulsive and OCD case-control score
P: p-value
PBon: p-value after Bonferroni correction
Q: Heterogeneity
RPTOR: Regulatory Associated Protein of mTOR, complex 1
SDQ: Strengths and Difficulties Questionnaire
SE: Standard Error
SEM: Structural Equation Modelling
SNP: Single Nucleotide Polymorphism
SRMR: Standardized Root Mean square Residual
STD: Standard Deviation
T2D: Type 2 Diabetes Mellitus
2hGlu: Glucose levels 2 hours after an oral glucose challenge

## 1 Introduction

Psychiatric conditions, like Attention-deficit/hyperactivity disorder (ADHD), autism spectrum disorder (ASD), and obsessive-compulsive disorder (OCD) are known to be heritable and have complex multifactorial etiologies (Faraone et al., 2019; Mahjani et al., 2021; Tick et al., 2016). Co-morbidity is highly prevalent in individuals with psychiatric disorders, which extends beyond psychiatric phenotypes and also includes insulin resistance (IR)-related somatic conditions, like type 2 diabetes mellitus (T2D) and obesity (Instanes et al., 2018; Isomura et al., 2018; Shedlock et al., 2016).

Many symptoms and characteristics of psychiatric disorders are also observed in healthy individuals. Such disorder-related traits tend to show a nearly normal distribution in the general population, with psychiatric cases clustering at the extreme end (Bralten et al., 2018). It was shown that this trait-disorder continuum also holds at the genetic level, demonstrated by genetic overlap between obsessive-compulsive traits (‘guilty taboo thoughts’) and OCD (Bralten et al., 2020; Burton et al., 2021), autistic-like traits (including reduced sociability) and ASD (Bralten et al., 2018, 2021) and attention-deficit/hyperactivity symptoms and ADHD (Middeldorp et al., 2016). Additionally, some of these traits span multiple diagnoses, indicating trans-diagnostic characteristics, which aligns well with genetic correlations between multiple psychiatric conditions found in cross-disorder studies (Cross-Disorder Group of the Psychiatric Genomics Consortium, 2019). With classical genetic case-control comparisons being challenged by the extent of heterogeneity and comorbidity within and across psychiatric and somatic conditions, exploring continuous phenotypes provides ample opportunities to help elucidate the observed heterogeneity in psychiatry and explore genetic links to IR-related somatic conditions (Insel et al., 2010; Taylor et al., 2019).

One connection between psychiatric conditions and IR-related somatic conditions might lie in dysregulated insulin signaling. Insulin has important functions in the brain (Chen et al., 2022), as it activates insulin receptors expressed on the surface of neuronal and glial cells, initiating a cascade of intracellular transduction processes (Pomytkin et al., 2018). By mediating cellular metabolism, insulin contributes to the development and homeostasis of the central nervous system, influencing neurogenesis, neuronal differentiation and promoting neurite growth (Agrawal et al., 2021). Additionally, insulin has protective properties in the brain preventing damage from apoptosis and oxidative stress (Agrawal et al., 2021). While metabolic disturbances in psychiatry have been perceived as possible consequences of unhealthy lifestyles, sedentary habits, or the chronic use of psychotropic medication (Grajales et al., 2019), the observation that glycemic and metabolic imbalances have been found in drug-naïve acute psychiatric patients already at disorder onset suggests the potential involvement of shared biological mechanisms (Coello et al., 2019). Epidemiological work shows that having T2D or other metabolic conditions during pregnancy increases the risk of the offspring developing ASD or ADHD (Chen et al., 2023; Krakowiak et al., 2012; Xiang et al., 2015). Furthermore, large-scale registry data convincingly showed that obesity is a relevant risk factor not only for developing T2D and metabolic syndrome, but also for receiving mental health diagnoses (Leutner et al., 2023) and that bidirectional associations exist between T2D and psychiatric disorders, including ADHD, ASD, and OCD (Wimberley et al., 2022). The observed multimorbidity between IR-related conditions and psychiatric disorders complicates clinical trajectories (Skou et al., 2022) and has been linked to more severe clinical outcomes (Fanelli et al., 2022a; Possidente et al., 2023), indicating the potential clinical relevance.

Some studies have indicated genetic links between psychiatric conditions and insulin signaling. In the case-control genetic comparisons of OCD, genes regulating insulin signaling were enriched in the top results (van de Vondervoort et al., 2016) and insulin-related traits, including levels of fasting insulin and 2 h glucose measures, showed significant genetic correlations with OCD (Bralten et al., 2020). A recent study investigated the genetic cross-links between nine neuropsychiatric disorders and three IR-related somatic diseases using publicly available genetic association data (Fanelli et al., 2022b). Their results indicate that the genetics of IR-related conditions may exert divergent pleiotropic effects, with OCD as well as anorexia nervosa and schizophrenia showing negative genetic overlap with somatic IR-related conditions, while ADHD and major depressive disorder showed positive genetic overlap with IR-related conditions (Fanelli et al., 2022b). A later study showed that the genetic correlations between ADHD, major depressive disorder, OCD and anorexia nervosa with three IR-related somatic diseases could be captured by a latent shared genetic factor that associated to an insulin binding gene-set (Mota et al., 2026). Next to global genetic correlations, also extensive local genetic correlations between ASD, OCD and ADHD with IR-related conditions have been reported (Fanelli et al., 2025). In addition, a recent family-based study was able to show that relatives of individuals with a psychiatric disorder had an increased risk for T2D and, within a subpart of their study, that genetic risk for T2D was associated with an increased risk for several psychiatric disorders, including ASD and ADHD (Wimberley et al., 2024). These studies highlight multiple genetic links between IR-related somatic diseases and ADHD, ASD, and OCD, but remain mainly bound by bivariate comparisons.

Genomic Structural Equation Modelling (Genomic SEM) is a multivariate method that can analyze the joint genetic architecture of complex traits (Grotzinger et al., 2019). First results indicate patterns of substructures in the genetic correlations between major psychiatric disorders and show that multivariate genetic analyses are able to find additional genetic risk variants that were not found in univariate analyses (Grotzinger et al., 2022, 2023). In this study, multivariate genomic SEM was applied to examine potential joint genetic architectures of trait-based genome-wide association studies (GWASs) focusing on the continuum of traits related to ADHD, ASD, and OCD, rather than the differences between cases and controls, in combination with IR-related measures. Subsequently, multivariate genetic analyses were performed on the modeled genetic factors to uncover genetic and biological underpinnings of the latent factors representing shared genetics between psychiatric and IR-related traits.

## 2 Material and methods

### 2.1 Data selection

Genetic association data for psychiatric and IR-related traits were selected based on availability, sample size, and relevance. The inclusion criteria for the summary statistics of GWASs consisted of a minimum sample size of 10,000 and having significant SNP-based heritability (p < 0.05; Z > 2). To ensure the inclusion of the largest possible GWAS datasets available, in addition to literature searches, the online database GWAS Catalog (Sollis et al., 2023) was utilized for the final selection.

### 2.2 GWASs of psychiatric traits

To include a continuous trait measure for ADHD, the GWAS meta-analysis of a total score of attention and hyperactivity/impulsivity symptoms in children (Middeldorp et al., 2016) was included. This meta-analysis included 17,666 children from nine population-based cohorts of the EArly Genetics and Life course Epidemiology (EAGLE) consortium. Data were based on scaled questionnaires, including the Attention Problems scale of the Child Behavior Checklist (CBCL) and the Hyperactivity scale of the Strengths and Difficulties Questionnaire (SDQ), consisting of parent- and teacher-rated scales (Middeldorp et al., 2016).

Previous GWASs of obsessive-compulsive symptoms did not reach the sample size requirements for Genomic SEM. Therefore, a GWAS that meta-analyzed obsessive-compulsive symptoms (OCS) in combination with OCD case-control status (Smit et al., 2020) was used. The OCS score in this meta-analysis used subscales that measured obsessions (rumination and impulsions) and compulsions (checking, washing, and ordering/precision), based on the Padua Inventory scale (Smit et al., 2020). The continuous OCS scores were measured in a population with an age range between 18 and 80 years, including 8267 individuals based on self-reported items. The OCD case-control subsample included 2688 individuals with OCD and 7037 controls from the Psychiatric Genomics Consortium OCD GWAS (International Obsessive Compulsive Disorder Foundation Genetics Collaborative (IOCDF-GC) and OCD Collaborative Genetics Association Studies (OCGAS).).

For ASD-related traits, no total symptom score GWAS reached the required sample size, therefore a GWAS on sociability (Bralten et al., 2021) was used, since social difficulties are at the core of the autistic phenotype and sociability and ASD show genetic overlap (Bralten et al., 2021). The sociability GWAS was performed on an aggregated score based on four questions related to social behavior in a total of 342,461 adults (mean age 56.61). In our analysis, the direction of beta association values of SNPs from the sociability GWAS summary statistics was reversed to indicate reduced sociability. An overview of GWAS datasets used is provided in Table 1.

**Table 1:** Overview of included GWAS datasets.

Phenotype	N	Study	PMID
ADHD total symptom score	17,666	Middeldorp et al., 2016	27663945
OCS + OCD	17,992	Smit et al., 2020	31891238
Sociability	342,461	Bralten et al., 2021	34054130
2hGlu	63,396	Chen et al., 2021	34059833
BMI	697,734	Pulit et al., 2019	30239722
FPG	140,595	Lagou et al., 2021	33402679
FPI	98,210	Lagou et al., 2021	33402679
HbA1c	123,665	Wheeler et al., 2017	28898252
HOMA-IR	37,037	Dupuis et al., 2010	20081858
HOMA-B	36,466	Dupuis et al., 2010	20081858

All included GWAS datasets were from analyses performed in samples with European ancestry. Abbreviations: ADHD = attention-deficit/hyperactivity disorder; OCD = obsessive-compulsive disorder; OCS = obsessive-compulsive symptoms; 2hGlu = glucose levels 2 hours after an oral glucose challenge; BMI = body mass index; FPI = fasting insulin; FPG = fasting glucose; HbA1c = glycated haemoglobin; HOMA-B = homeostatic model assessment of β-cell function; HOMA-IR = homeostatic model assessment for insulin resistance.

### 2.3 GWASs of IR-related traits

GWAS data on IR-related traits were preselected based on the aforementioned inclusion criteria. To guide our selection for relevance to ADHD, ASD and OCD, the included IR-related traits were considered traits that genetically correlate with at least one of the psychiatric conditions in a bivariate way (see Supplementary Materials in Supplementary Fig. S1 and Table S1), leading to the selection of five IR-traits: glucose levels 2 h after an oral glucose challenge (2hGlu), body mass index (BMI), fasting glucose (FPG), fasting insulin (FPI), glycated haemoglobin (HbA1c). Two IR-related traits did not reach significance: homeostatic model assessment for insulin resistance (HOMA-IR), and homeostatic model assessment of β-cell function (HOMA-B) (Agrawal et al., 2021; Chen et al., 2021; Dupuis et al., 2010; Lagou et al., 2021; Pulit et al., 2019; Wheeler et al., 2017). An overview of GWAS datasets used is provided in Table 1.

### 2.4 Genomic structural equation modeling

Genomic SEM was applied to investigate latent genetic factors underlying the three psychiatric trait GWASs (attention-deficit/hyperactivity symptom scores, OCD + OCS symptoms, reduced sociability) and the five IR-related trait GWASs (BMI, FPG, FPI, HbA1c, HOMA-IR). Genomic SEM is a data-driven, multivariate genetic analysis approach used to identify genetic latent factors underlying different variables, that accounts for sample overlap and sample size variability of the input datasets (Grotzinger et al., 2019). The analysis involves exploratory and confirmatory factor analyses (EFA and CFA, respectively) to determine the model based on loading thresholds above 0.25 and examines model fit. Factor analyses were performed separately for the odd and even chromosomes to avoid overfitting. In the first step of Genomic SEM, the genetic covariance matrix and sampling covariance matrix were estimated using precomputed linkage disequilibrium (LD) scores obtained from the 1000 Genomes Project (Genomes Project et al., 2015). During the second step, the model fit was estimated using standardized root mean square residual (SRMR), model χ2, Akaike Information Criterion (AIC), and the Comparative Fit Index (CFI). The model fit statistic CFI tests to what degree the proposed model fits compared to a model in which all traits are heritable but not correlated genetically (>0.95 is considered good fit), and AIC is a relative fit statistic to compare models, with lower values representing a better fit (Grotzinger et al., 2019). The analysis followed procedures described on the Genomic SEM GitHub, using default parameters (see https://github.com/GenomicSEM/GenomicSEM).

### 2.5 Multivariate GWAS

Multivariate GWASs were performed within the Genomic SEM framework on the factors that loaded both psychiatric and IR-related traits to estimate individual SNP effects on the latent factors. Multivariate GWAS was run on SNPs filtered on minor allele frequency (MAF) (MAF>0.01) to ensure that effect estimates are based on enough individuals and quality info score (INFO>0.6) to ensure genotyping quality, when such information was available (Grotzinger et al., 2022). SNPs were considered significant if they reached the genome-wide significance threshold of p < 5e-08. A SNP-level heterogeneity test (Q_SNP_) was then performed to test the null hypothesis that a SNP acts on the latent factor instead of acting on individual or subsets of traits/observed variables. Therefore, the heterogeneity (Q) index (Grotzinger et al., 2019, 2022) was calculated by using independent pathway models in which the effects of the SNPs on the traits and the residual variances are freely estimated. The common and independent pathway models are then used to estimate a χ2 distributed Q_SNP_ test statistics with degrees of freedom (df) equal to k−1, where k reflects the number of included phenotypes. Since we are interested in understanding the shared genetic basis of the latent factors, significant Q_SNP_s (p < 5e −08) were removed for subsequent analyses as these SNPs do not represent direct effects on latent factors. The effective sample size (N_eff_) for each psychiatric-IR factor was calculated (Grotzinger et al., 2019; Mallard et al., 2022) and in this calculation the summary statistics are restricted to MAF limits of 10% and 40% to produce stable estimates, as described on the Genomic SEM Github (https://github.com/GenomicSEM/GenomicSEM/wiki/5.-User-Specified-Models-with-SNP-Effects).

### 2.6 FUMA

The online tool for functional mapping and annotation of GWASs (FUMA v1.5.4) (Watanabe et al., 2017) was utilized to interpret the multivariate GWAS outcomes of each psychiatric-IR related latent factor. The SNP2GENE module within FUMA was used to perform gene mapping (i.e., positional mapping, eQTL mapping), as well as gene-based, gene-property, and gene set analyses in Multi-marker Analysis of GenoMic Annotation (MAGMA). The gene-wide genome-wide significance threshold was set at p = 0.05/16,888 = 2.961e-06 accounting for the 16,888 tested protein coding genes. The gene set analysis was performed on 5917 Gene Ontology (GO) terms and 4761 curated gene sets obtained from MsigDB v6.2. Gene set analysis results were considered significant at p < 0.05/10,678 = 4.68e-06. For the gene-property analyses 30 GTEx/v8, 53 GTEx/v8 and 13 BrainSpan gene expression datasets were selected to examine gene expression in brain-related regions in adults and during brain development. As input for the sample size parameter (N) in FUMA the N_eff_ was used.

## 3 Results

### 3.1 Identification of psychiatric-IR latent genetic factors

**Fig. 1:**
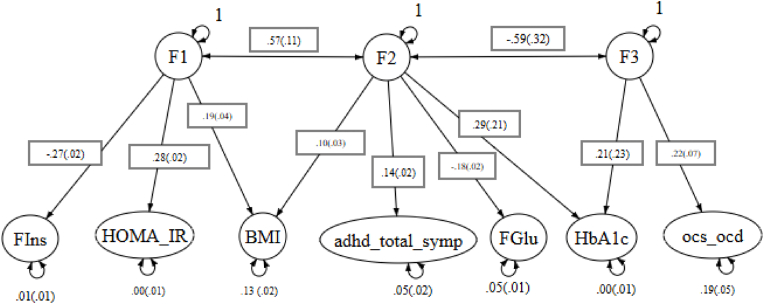
Three-factor model path diagram**.** The structural equation model path diagram of the identified three factor model as the best model fit to the data based on the confirmatory factor analysis in Genomic SEM with standardized parameter estimates. Abbreviations: IR=Insulin Resistance, ADHD_total_symp = ADHD total symptom score; OCS_OCD = obsessive-compulsive symptoms and OCD case-control score; BMI = body mass index; FPI = fasting insulin; FPG = fasting glucose; HbA1c = glycated haemoglobin; HOMA-IR = homeostatic model assessment for insulin resistance. Figure created with https://semdiag.psychstat.org/.

Based on our input data of eight variables (three psychiatric traits, ADHD total symptom score, OCS and OCD case-control score, reduced sociability and five IR-related continuous traits, BMI, FPI, FPG, HbA1c, HOMA-IR) four potential models were provided by the EFA analysis within Genomic SEM on odd chromosomes. The subsequent CFA analysis on these models on even chromosomes confirmed the best model fit for a three-factor model (χ2(df = 9) = 22.87, AIC = 60.8, CFI = 0.97, SRMR = 0.093), see Fig. 1. This model also fits well for all chromosomes (χ2(df = 9) = 18.79, AIC = 56.8, CFI = 0.99, SRMR = 0.068). Details on robustness, model fit statistics and standardized factor loadings of this model can be found in Supplementary Tables S4–S7.

The first factor was loaded solely by IR-related traits (BMI, FPI, and HOMA-IR). The second factor included ADHD total symptom scores together with BMI, FPG, and HbA1c, which we named the ADHD-IR factor. The third factor included OCD + OCS together with HbA1c, which we named the OCD/OCS-IR factor. Reduced sociability did not load to any of the factors.

### 3.2 SNP associations

Multivariate genome-wide association analyses were performed on the ADHD-IR and the OCD/OCS-IR factors. Heterogeneity tests indicated a total of 1304 heterogeneous Q_SNP_s for the ADHD-IR factor and 813 heterogeneous Q_SNP_s for the OCD/OCS-IR latent factor, which were removed from subsequent analyses. No genome-wide significant SNPs were found (see Supplementary Figs. S2 and S3), the most significant SNPs for the ADHD-IR factor and the OCD/OCS-IR factor provided by FUMA can be found in Supplementary Tables S2 and S3. Additional quality control statistics (quantile-quantile plots) are available in Supplementary Figs. S4 and S5. The summary statistics of the genomic factors can be found here: DOI 10.34973/sya1-b485.

### 3.3 Gene-level associations

**Fig. 2:**
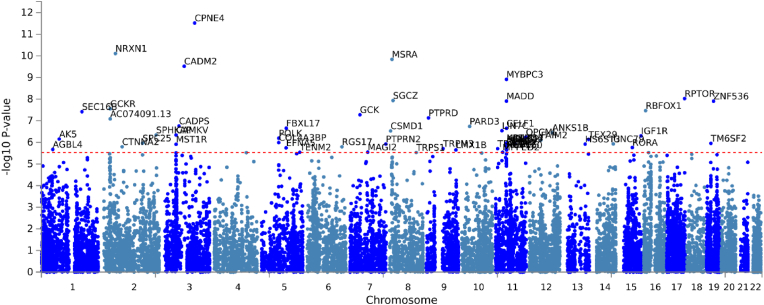
Manhattan plot of the gene-based analysis of the ADHD-IR latent factor. Gene-based Manhattan plot based on the input GWAS summary statistics of the multivariate GWAS of the ADHD-IR factor. The input SNPs were mapped to a total of 16,888 protein coding genes. The x-axis displays the positional location on the different chromosomes, and the y-axis displays the −log p-value of the gene-wide associations. The genome-wide significance threshold (red dashed line in the plot) was set at p = 0.05/16,888 = 2.961e-6 accounting for the number of genes tested.

**Fig. 3:**
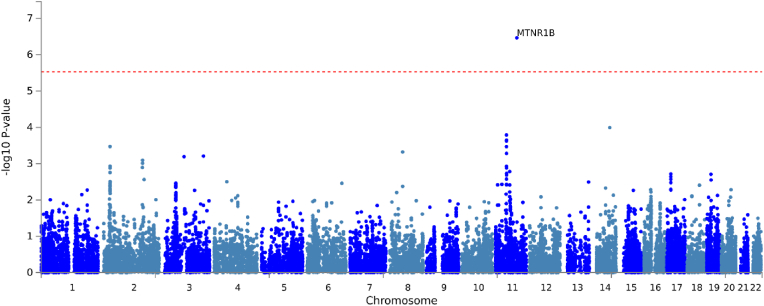
Manhattan plot of gene-based analysis of the OCD/OCS-IR latent factor. Gene-based Manhattan plot based on the input GWAS summary statistics of the multivariate GWAS of the OCD/OCS-IR latent factor. The input SNPs were mapped to a total of 16,888 protein coding genes. The x-axis displays the positional location on the different chromosomes, and the y-axis displays the −log p-value of the gene-wide associations. The genome-wide significance threshold (red dashed line in the plot) was set at p = 0.05/16888 = 2.961e-6. accounting for the number of genes tested.

Using the FUMA SNP2GENES module, SNPs from the multivariate GWAS output of the ADHD-IR and the OCD/OCS-IR latent factors were annotated to 16,888 protein coding genes. The built-in MAGMA gene-based analysis identified 57 genes reaching genome-wide significance for association with the ADHD-IR factor (Fig. 2 and Supplementary Table S4), and one gene, *MTNR1B* (p = 3.4364e-07), for association with the OCD/OCS-IR factor (Fig. 3).

### 3.4 Gene set enrichment and tissue specificity

Subsequently, with MAGMA gene-set analysis we explored factor-associated gene sets. This analytical step yielded significant results for 12 gene sets associated with the ADHD-IR factor and five gene ontology terms associated with the OCD/OCS-IR factor (Table 2, Table 3, respectively), including neuron differentiation, neuron development and neurogenesis.

**Table 2:** Results of the MAGMA gene set based analysis of the ADHD-IR latent factor. The table shows the top significant identified gene sets based on Bonferroni corrected p-values accounting for 5917 Gene Ontology (GO) terms and 4761 curated gene sets tested. N genes = number of genes; Beta = beta value; Beta STD = standard deviation of the beta value; SE = standard error; P = p-value; PBon = p-value after Bonferroni correction.

Gene set	N genes	Beta	Beta STD	SE	P	PBon
Neuron_differentiation	1162	0.16823	0.042594	0.029012	3.4126e-09	5.28202228e-05
Neuron_development	956	0.16921	0.039113	0.031859	5.5211e-08	0.000854500647
Neurogenesis	1383	0.14199	0.038943	0.02681	5.9978e-08	0.000928219528
Positive_regulation_of_biosynthetic_process	1692	0.1206	0.036219	0.02396	2.4373e-07	0.00377172175
Negative_regulation_of_signaling	1159	0.13615	0.034429	0.028454	8.6411e-07	0.01337123814
Positive_regulation_of_gene_expression	1669	0.11499	0.034324	0.024369	1.1983e-06	0.0185412959
Dna_binding_transcription_factor_activity	1443	0.13257	0.037068	0.028133	1.2356e-06	0.0191172032
Negative_regulation_of_biosynthetic_process	1272	0.12736	0.03362	0.027117	1.3335e-06	0.0206305785
Cellular_response_to_endogenous_stimulus	1183	0.13286	0.033918	0.028819	2.0294e-06	0.031394818
Regulation_of_nervous_system_development	790	0.16366	0.034567	0.035686	2.2771e-06	0.0352244599
Signal_release	408	0.22339	0.034309	0.048843	2.4156e-06	0.0373645008
Positive_regulation_of_rna_biosynthetic_process	1378	0.12058	0.033017	0.026425	2.5407e-06	0.0392970069

**Table 3:** Results of the MAGMA gene set based analysis of the OCD/OCS-IR latent factor. The table shows the top significant identified gene sets based on Bonferroni corrected p-values accounting for 5917 Gene Ontology (GO) terms and 4761 curated gene sets tested. N genes = number of genes; Beta = beta value; Beta STD = standard deviation of the beta value; SE = standard error; P = p-value; PBon = p-value after Bonferroni correction.

Gene set	N genes	Beta	Beta STD	SE	P	PBon
Positive_regulation_of_rna_biosynthetic_process	1378	0.097151	0.026599	0.019947	5.6251e-07	0.00870652978
Dna_binding_transcription_factor_activity	1444	0.10013	0.028003	0.021234	1.2167e-06	0.0188308659
Neuron_differentiation	1162	0.10139	0.025669	0.021905	1.8554e-06	0.0287141704
Nucleoplasm_part	957	0.10355	0.023944	0.022468	2.0439e-06	0.0316293525
Positive_regulation_of_gene_expression	1669	0.084632	0.02526	0.018406	2.1496e-06	0.0332629104

Gene-property analyses indicated significant enrichments in the brain and pituitary for genes related to the ADHD-IR factor across 30 general tissue types. Moreover, it indicated significant enrichments in almost all tested brain regions, including the cerebellum, except the substantia nigra for genes related to the ADHD-IR factor across 53 tissue types. Additionally, significant enrichments were found between early and late mid-prenatal developmental stages of the brain for genes related to the ADHD-IR factor (Supplementary Fig. S6). For the OCD/OCS-IR factor, significant enrichments were found in the brain, pituitary and uterus across 30 general tissue types and seven out of the 13 brain regions across 53 specific tissue types. In addition, significant enrichments were observed from early prenatal to late mid-prenatal developmental stages of the brain for genes related to the OCD/OCS-IR factor (Supplementary Fig. S7).

## 4 Discussion

Employing multivariate genomic analyses this study demonstrates the existence of latent genetic factors that include both psychiatric as well as IR-related traits, indicating shared etiologies between them. These results suggest that phenotypic links between psychiatric conditions and IR-related conditions are not purely driven by disorder status, and that genetic studies can help to find relevant underlying biological mechanisms. Follow-up bioinformatic analyses of the genetic factors that included both psychiatric as well as IR-related traits indicate the potential involvement of IR-related genes, genes involved in neuron differentiation, neuron development, and neurogenesis and enrichment for expression in the brain.

One latent genetic factor was loaded by ADHD total symptom scores and three IR-related traits, BMI, FPG, and HbA1c, in line with previously reported positive genetic correlations of these IR-related traits with ADHD (Fanelli et al., 2022b). Results are also in line with phenotypic observations of elevated HbA1c levels in children with ADHD compared to children without ADHD (Lindblad et al., 2015), and observations that children with ADHD show increased waist circumference and BMI compared to children without ADHD, with these measures being linked to the severity of the condition (Salvat et al., 2022). In addition, poor glycemic control within diabetic patients has been linked to increased risk for ADHD (Liu et al., 2021) and individuals diagnosed with T2D are susceptible to presenting ADHD-related symptoms (Dehnavi et al., 2023). A noticeable mechanistic connection is the link between insulin signaling and dopamine signaling in the midbrain, relevant for reward behavior (Gruber et al., 2023). Altered reward sensitivity is a known feature in ADHD (Luman et al., 2010) and dopamine and ADHD have been linked through multiple routes, including genetics (Bralten et al., 2013), brain imaging (Grimm et al., 2022), and the pharmacodynamics of symptom reducing medications (Faraone, 2018).

The genetic associations to the ADHD-IR factor point to several genes that are important for both metabolic processes as well as brain processes. The *CPNE4* gene encodes for calcium-dependent membrane binding proteins enriched in neurons (Goel et al., 2021) and is involved in insulin secretion and glucose uptake regulation (El-Huneidi et al., 2021). Another top gene was *CADM2,* previously linked to core ADHD features like impulsivity and risk-taking behavior (Sanchez-Roige et al., 2023). Conditional genetic analyses showed that variants within *CADM2* influence both psychiatric and metabolic traits, including BMI, and are expressed in adult brain and adipose tissue (Morris et al., 2019). Interventions on expression of *CADM2* in animal models resulted in changes in adiposity, systemic glucose levels and insulin sensitivity (Yan et al., 2018), linking this gene to glycemic regulation. Another top gene *NRXN1*, is implicated in insulin vesicle granule docking (Mosedale et al., 2012) and psychiatric disorders, including ASD and depression (Hughes et al., 2022; Skiba et al., 2021; Sudhof, 2017). An ASD mouse model with reduced Nrxn1α expression showed increased glucose metabolism in the dorsal raphe nucleus, and decreased insulin receptor signaling in the prefrontal cortex, a top identified brain region in our gene property analysis (Sudhof, 2017). The *RPTOR* gene was also found, a gene that encodes the regulatory-associated protein of mTOR, or raptor, implicated in the mTOR pathway and regulating cell growth and survival (Debniak et al., 2014). This regulatory protein is sensitive to insulin levels, and also regulates glucose metabolism and synaptic plasticity via the mTOR cascade (Grunblatt et al., 2023). Genetic variants within the mTOR pathway have been described as a link between brain volume and ASD in earlier analyses (Arenella et al., 2023), and upregulation of mTOR-related genes has not only been observed in ASD, but also in ADHD (Grunblatt et al., 2023). While the current study focused on traits, top genes like *SEC16B* and *RGS17* overlap with genes reported in multivariate genetic analyses that included psychiatric conditions and IR-related conditions (Mota et al., 2026). Reported gene set analyses included associations with neuron differentiation, neuron development, and neurogenesis in accordance with ADHD being a neurodevelopmental disorder (Vaidya, 2012). Based on accumulating evidence for the actions of insulin in the brain (Agrawal et al., 2021; Chiu et al., 2010), one could speculate that the role of insulin in neurodevelopment could be important in relation to our identified factor. In tissue expression analyses of the genes associated with the ADHD-IR latent factor, significant enrichments were observed in all tested brain regions except the pituitary and the substantia nigra. The positive association between gene expression in the cerebellum and genetic associations of the ADHD-IR factor link to the finding of a previous study reporting an association between smaller volume of the vermis of the cerebellum and the amount of ADHD symptoms (Ivanov et al., 2014). ADHD symptom scores on an attention scale also associated with smaller surface areas, including frontal gyrus and total surface area, in a pediatric population cohort (Hoogman et al., 2019). Our findings linking gene expression in the frontal cortex, BA9 region and brain cortex are in line with previous work that suggest that aberrated maturation of frontal lobes may result in ADHD symptoms in a subgroup of children with ADHD (Leisman et al., 2022).

A latent genetic factor loading OCD/OCS and HbA1c was also observed. An IR-related OCD/OCS factor is in line with studies linking insulin to OCD. Among these, Hou and colleagues found that individuals diagnosed with OCD present increased glucose metabolism in the orbitofrontal cortex compared to healthy controls (Hou et al., 2022), aligning with rodent models of T2D that presented compulsivity-related behavior and increased glucose levels in the dorsomedial striatum (van de Vondervoort et al., 2019). Also large genetic analyses of OCD case-control comparisons indicated insulin genes in their top results (van de Vondervoort et al., 2016), which was also found for genetic analyses of OCS (Bralten et al., 2020). For HbA1c no genetic sharing with OCD measures was observed previously (Bralten et al., 2020). Nonetheless, HbA1c levels have been shown to be positively correlated to OCD symptomatology (Kontoangelos et al., 2013). Prior work did report genetic sharing between OCD and FPI levels and the 2hGlu measure (Bralten et al., 2020), while negative genetic correlations of BMI and obesity have been described with OCD diagnosis (Fanelli et al., 2022b). Strom and colleagues defined OCD subgroups based on comorbid diagnoses and genetic relations between psychiatric disorders with somatic and mental measures. Their study showed that polygenic scores for BMI were more negatively associated with the OCD group without comorbidities compared to OCD subgroups having a comorbid condition, such as ADHD (Strom et al., 2021). These results might be in line with disparate genetic relationships of OCD versus ADHD to different IR-related traits, denoting potential different underlying mechanisms. This is in line with previously reported genetic clusters related to IR-related multimorbidity, placing ADHD and OCD in distinct clusters (Fanelli et al., 2022b).

The *MTNR1B* gene was identified as the only significant genome-wide significant gene associated with the OCD/OCS-IR factor. This gene encodes the melatonin receptor 1B, a high affinity membrane receptor for the melatonin hormone (Lane et al., 2016). Elevated melatonin levels may impair glucose tolerance and modulate fasting glucose levels (Hu et al., 2010), linking this gene to glycemic regulation. Altered levels of melatonin have been associated with the risk for type 2 diabetes and disturbed circadian rhythm (Lane et al., 2016; Wu et al., 2022). Lane and colleagues investigated circadian rhythm based on sleep measures via self-reports and physiological measurements with polysomnography. They reported that circadian disruption may mediate risk for type 2 diabetes via *MTNR1B* (Lane et al., 2016). Sleep disturbances are also described in OCD, linking the severity of obsessive-compulsive symptoms to sleep quality (Segalas et al., 2021) and highlighting sleep disturbances as a shared symptom between OCD and IR-related conditions. Positive associations between the mid-prenatal and prenatal developmental stages of the brain and the genetic associations of the OCD- IR-related factor were reported, linking the OCD/OCS-IR genetic factor to fetal brain development. A similar conclusion was reached regarding OCD and fetal origin based on BrainSpan transcriptome profiles in a supervised learning approach study (Jia et al., 2021), also supporting the link of prenatal developmental trajectory to our genetic factor.

A potential role of insulin signaling in ASD was hypothesized, but no genetic factor including reduced sociability as a proxy for ASD-related symptoms and the IR-related traits was found. Rather than concluding no genetic link, we must acknowledge that the method used here is based on global genetic correlation matrices, and previous work also did not find global or local genetic correlations between IR-related measures and ASD (Fanelli et al., 2022b, 2025), while stratified analyses did indicate a significant covariance for genes within the insulin signaling pathway between ASD and metabolic syndrome (Fanelli et al., 2022b), indicating that the genetic relationships between IR and ASD might be more localized, making global genetic correlation methods not the best fit. In addition, an ASD diagnosis requires more than reduced sociability-related symptoms, but the current analyses were restricted to genetic data with adequate sample size and limited by the absence of a well-powered GWAS for other ASD symptoms. Therefore we cannot exclude the possibility that the current null finding for our specific ASD trait-proxy may reflect phenotype mismatch rather than biological absence of overlap.

This study has several strengths and limitations. By exploring the largest genetic datasets in the world and moving beyond traditional case-control comparisons, novel insights into the biological basis of symptom dimensions in psychiatry and their link to insulin resistance-related traits were gained. However, as the analyses rely on publicly available GWAS data, not all traits of interest could be studied, which may impede the discovery of relevant genetic clusters of variants. Although in this manuscript insulin signaling is discussed as a potential shared biological process between psychiatric conditions and IR-related conditions, the current trait-based analyses are based on association data restricting any claims on causality. Additionally, the use of exclusively European cohorts hinders the generalizability of our findings to other ethnicities. Future research should examine if the genetic clustering can be observed in diverse populations.

## 5 Conclusions

In conclusion, this study identified latent genetic factors characterized by shared liabilities underlying ADHD symptoms with IR-related measures and obsessive-compulsive symptoms and OCD with an IR-related measure. Revealing shared genetics between psychiatric-like traits and glycemic measures in population-based data demonstrates that the link between insulin signaling and psychiatry is not solely driven by disorder status. These insights may pave the way for IR-focused clustering approaches within psychiatric disorders like ADHD and OCD that could lead to subgroups that are characterized by specific IR-related profiles.

## Ethical statement

We utilized publicly available summary statistics of genome-wide association studies conducted by external consortia and studies, and therefore no authorization was required from the local Ethics Committee.

## Declaration of competing interest

The authors declare the following financial interests/personal relationships which may be considered as potential competing interests:Janita Bralten reports financial support was provided by Dutch Research Council (NWO) Health Research and Development (ZonMW). Nina Roth Mota reports financial support was provided by the National Institute Of Mental Health of the National Institutes of Health. Janita Bralten reports equipment, drugs, or supplies was provided by SURF Cooperative. Janita Bralten reports financial support was provided by European Union Horizon 2020 research and innovation program. Marieke Klein reports financial support was provided by Dutch Research Council (NWO) Health Research and Development (ZonMW). If there are other authors, they declare that they have no known competing financial interests or personal relationships that could have appeared to influence the work reported in this paper.

## Data Availability

Summary statistics of the multivariate genomic model are available at DOI 10.34973/sya1-b485.
