# Supplementary material for: Shared genetic architecture between psychiatric and insulin-related traits in the general population
Source: Neurosci Appl. 2026 Jun 17;5:107017. doi: 10.1016/j.nsa.2026.107017 (PMC13312545; doi:10.1016/j.nsa.2026.107017)
Supplement: Multimedia component 1 [file mmc1.docx]

# **Supplementary Materials**

Linkage Disequilibrium Score Regression

To test genetic correlations for the inclusion criteria of our model we performed linkage disequilibrium score regression (LDSC) to obtain genetic correlations (rg) between the most recent ADHD GWAS (Demontis et al., 2023), ASD GWAS (Grove et al., 2019), and OCD GWAS (International Obsessive Compulsive Disorder Foundation Genetics & Studies, 2018) with the IR-related GWAS traits. The global genetic correlations were estimated as described on the publicly available GitHub page (https://github.com/bulik/ldsc) (Bulik-Sullivan et al., 2015). The LDSC analysis utilized linkage disequilibrium (LD) scores based on 1000 Genomes for GWASs of European descent (Genomes Project et al., 2015). The method involves two steps. First, the input summary statistics were munged to the appropriate LDSC format and filtered on info and MAF. Thereafter the genetic correlations are estimated. LDSC analysis to estimate genetic correlations was restricted to GWAS summary statistics with sample size N > 5 000. Given the exploratory phase of this study we included nominally significant genetic correlations in follow-up analyses. The observed genetic correlations between the aforementioned disorders and IR-related traits are summarized in Supplementary Table 1. We found nominally significant genetic correlations (p<0.05) for a total of five IR-related traits, namely BMI, FPG, FPI, HbA1c and HOMA-IR. In addition, compared to the study of Fanelli et al. (2022) we identified an additional nominally significant genetic correlation between ADHD and HOMA-IR (rg=0.1727, p=0.0218) based on the larger most recent ADHD GWAS.


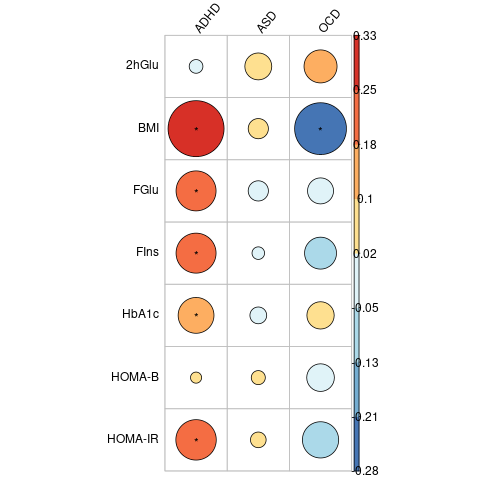


Fig. S1: Genetic correlation plot and trait flow diagram. The circle represents the level of genetic correlations and the colors show the direction of correlation (warmer colors indicating a positive direction and cooler colors indicating a negative direction). Abbreviations: ADHD= attention-deficit/hyperactivity disorder; ASD= autism spectrum disorder; OCD= obsessive-compulsive disorder; 2hGlu= glucose levels two hours after an oral glucose challenge; BMI=body mass index; FPI=fasting insulin; FPG=fasting glucose; HbA1c=glycated haemoglobin; HOMA-B=homeostatic model assessment of β-cell function; HOMA-IR=homeostatic model assessment for insulin resistance. *Nominally significant genetic correlation (p<0.05).

Table S1. Genetic correlations between ADHD, ASD, OCD with IR-related traits. Observed genetic correlations values (rg (p-value)). Abbreviations: ADHD= attention-deficit/hyperactivity disorder; ASD= autism spectrum disorder; OCD= obsessive-compulsive disorder; 2hGlu= glucose levels two hours after an oral glucose challenge; BMI=body mass index; FPI=fasting insulin; FPG=fasting glucose; Hb1ac=glycated haemoglobin; HOMA-B=homeostatic model assessment of β-cell function; HOMA-IR=homeostatic model assessment for insulin resistance. *Nominally significant genetic correlation (p<0.05).

| Disorder/trait | 2hGlu | BMI | FPG | FPI | HbA1c | HOMA-B | HOMA-IR |
| --- | --- | --- | --- | --- | --- | --- | --- |
| ADHD | -0.0199 (0.6621) | 0.3312 (1.0956e-63)* | 0.1691 (7.0e-4)* | 0.1691 (1.0e-4)* | 0.1356 (2.0e-4)* | 0.0131(0.8447) | 0.1727  ( 0.0218)* |
| ASD | 0.0766 (0.2263) | 0.0432 (1.6397e-01) | -0.0433 (0.3329) | -0.0167(0.7972) | -0.0298 (0.6188) | 0.0209(0.8516) | 0.0260 (0.8172) |
| OCD | 0.1152 (0.1678 ) | -0.2839 (2.5725e-11)* | -0.0716 (0.3398) | -0.1076 (0.1981) | 0.0787 (0.3670) | -0.0807(0.4817) | -0.1387 (0.2552) |


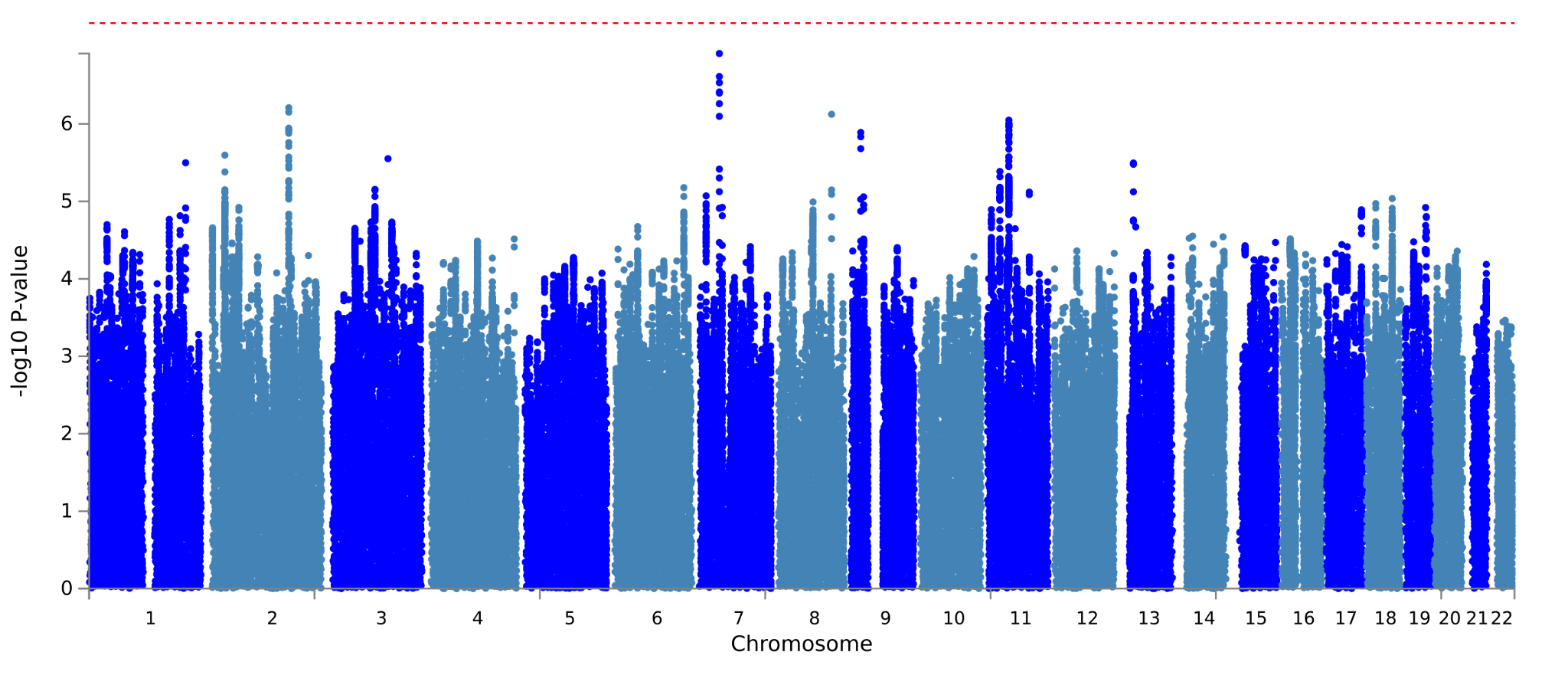


Fig. S2: Manhattan plot of GWAS summary statistics of ADHD-IR latent factor. The x-axis displays the chromosomes, and the y-axis displays the −log p-value. The red dashed line in the plot is the genome-wide significance threshold (p=5e-8).


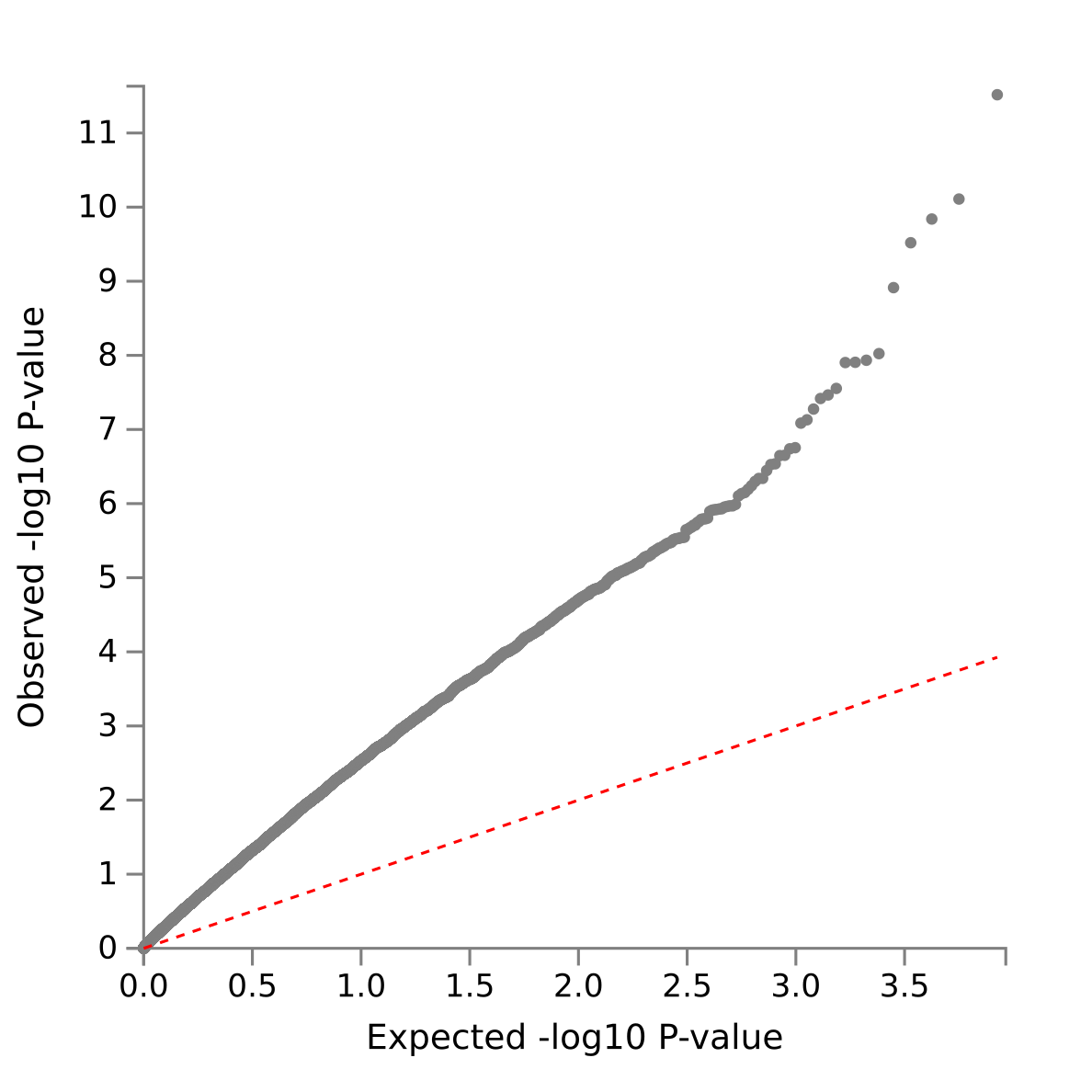


Fig. S4 Q-Q plots of ADHD-IR latent factor. Quantile-quantile (Q-Q) plot of GWAS summary statistics of ADHD-IR latent factor The x-axis displays the expected −log p-value, and the y-axis displays the observed p-value.

Table S2. Top 10 SNPs of GWAS summary statistics of ADHD-IR latent factor. Top 10 significant SNPs of the input GWAS summary statistics of ADHD-IR latent factor after removal of significant Q SNPs. Genome wide significance threshold was set at p=5e−8. chr=chromosome; bp=base pair position; non_effect_allele=non-effect allele; effect_allele=effect allele; rsID=variant ID; p=p-value; beta=beta value; se=standard error.


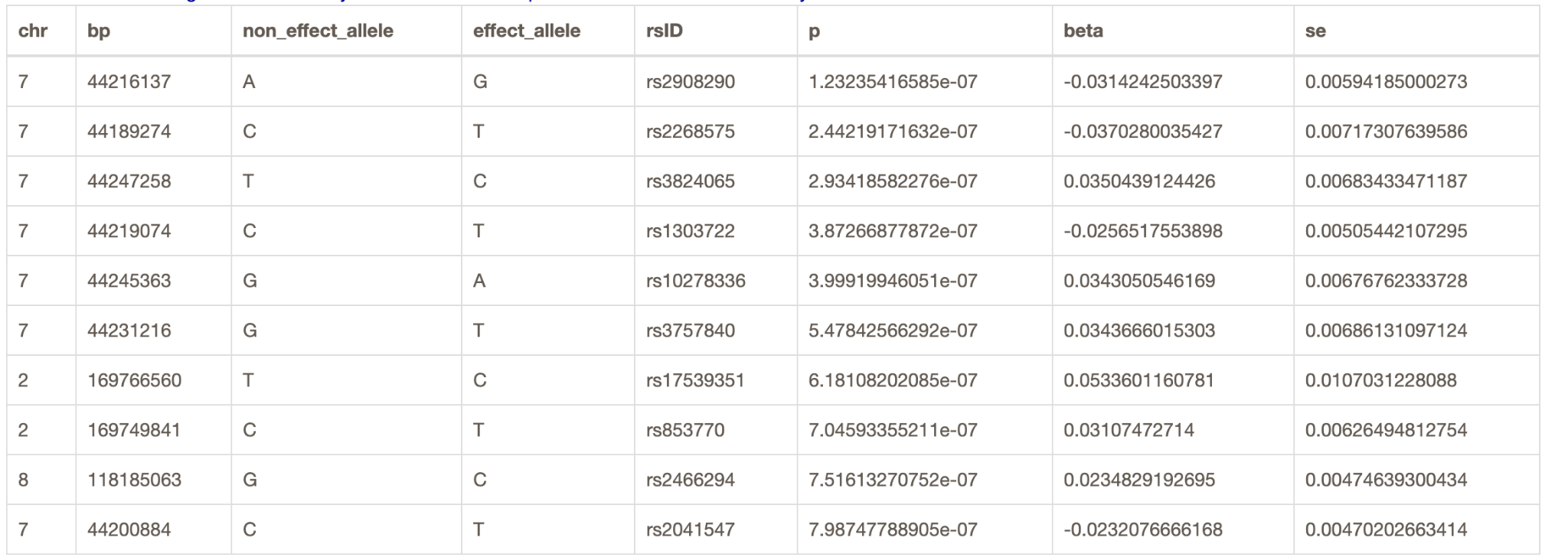


Table S4. Gene-based top significant associations results for the ADHD-IR latent factor. Genome wide significance threshold was set at p=0.05/16888=2.961e-6 accounting for the 16888 number of tested genes. CHR=chromosome; START=start position gene; STOP=end position of gene; NSNPS= number of SNPs; NPARAM=number of parameters; N=sample size; ZSTAT=Z-value for the gene based on its p-value; P=p-value.

| Ensembl ID | CHR | START | STOP | NSNPS | NPARAM | N | ZSTAT | P | GENE |
| --- | --- | --- | --- | --- | --- | --- | --- | --- | --- |
| ENSG00000196353 | 3 | 131252399 | 132004254 | 877 | 54 | 499492 | 6.8775 | 3.0464e-12 | CPNE4 |
| ENSG00000179915 | 2 | 50145643 | 51259674 | 1275 | 79 | 499492 | 6.3997 | 7.7826e-11 | NRXN1 |
| ENSG00000175806 | 8 | 9911778 | 10286401 | 562 | 37 | 499492 | 6.3043 | 1.4478e-10 | MSRA |
| ENSG00000175161 | 3 | 85008132 | 86123579 | 677 | 28 | 499492 | 6.1891 | 3.0255e-10 | CADM2 |
| ENSG00000134571 | 11 | 47352957 | 47374253 | 11 | 4 | 499492 | 5.9654 | 1.2199e-09 | MYBPC3 |
| ENSG00000141564 | 17 | 78518619 | 78940171 | 470 | 42 | 499492 | 5.6214 | 9.4726e-09 | RPTOR |
| ENSG00000185053 | 8 | 13947373 | 15095848 | 1484 | 98 | 499492 | 5.5857 | 1.164e-08 | SGCZ |
| ENSG00000110514 | 11 | 47290712 | 47351582 | 15 | 3 | 499492 | 5.5746 | 1.2404e-08 | MADD |
| ENSG00000198597 | 19 | 30719197 | 31204445 | 317 | 48 | 499492 | 5.5733 | 1.2501e-08 | ZNF536 |
| ENSG00000084734 | 2 | 27719709 | 27746554 | 7 | 2 | 499492 | 5.4315 | 2.7935e-08 | GCKR |
| ENSG00000078328 | 16 | 6069095 | 7763340 | 2640 | 260 | 499492 | 5.3952 | 3.4228e-08 | RBFOX1 |
| ENSG00000120341 | 1 | 177893091 | 177953438 | 74 | 14 | 499492 | 5.3754 | 3.8197e-08 | SEC16B |
| ENSG00000106633 | 7 | 44183872 | 44237769 | 26 | 7 | 499492 | 5.316 | 5.3049e-08 | GCK |
| ENSG00000153707 | 9 | 8314246 | 10612723 | 3112 | 282 | 499492 | 5.255 | 7.4016e-08 | PTPRD |
| ENSG00000205334 | 2 | 27928653 | 27938599 | 6 | 2 | 499492 | 5.2359 | 8.21e-08 | AC074091.13 |
| ENSG00000163618 | 3 | 62384022 | 62861054 | 503 | 56 | 499492 | 5.0931 | 1.7614e-07 | CADPS |
| ENSG00000148498 | 10 | 34398488 | 35104253 | 503 | 28 | 499492 | 5.0866 | 1.8228e-07 | PARD3 |
| ENSG00000145743 | 5 | 107194736 | 107717799 | 450 | 29 | 499492 | 5.0482 | 2.2296e-07 | FBXL17 |
| ENSG00000149187 | 11 | 47487496 | 47587121 | 21 | 5 | 499492 | 5.0463 | 2.2519e-07 | CELF1 |
| ENSG00000148943 | 11 | 27516123 | 27528320 | 3 | 1 | 499492 | 4.9976 | 2.9025e-07 | LIN7C |
| ENSG00000183117 | 8 | 2792875 | 4852494 | 4262 | 366 | 499492 | 4.9928 | 2.975e-07 | CSMD1 |
| ENSG00000185046 | 12 | 99120235 | 100378432 | 881 | 80 | 499492 | 4.9571 | 3.5782e-07 | ANKS1B |
| ENSG00000153820 | 2 | 228844666 | 229046361 | 170 | 18 | 499492 | 4.9094 | 4.5677e-07 | SPHKAP |
| ENSG00000164076 | 3 | 49895421 | 49907655 | 7 | 2 | 499492 | 4.9088 | 4.5809e-07 | CAMKV |
| ENSG00000140443 | 15 | 99192200 | 99507759 | 201 | 39 | 499492 | 4.8907 | 5.0252e-07 | IGF1R |
| ENSG00000183715 | 11 | 132284871 | 133402414 | 1094 | 146 | 499492 | 4.8646 | 5.7355e-07 | OPCML |
| ENSG00000122008 | 5 | 74807581 | 74896969 | 25 | 5 | 499492 | 4.8424 | 6.4139e-07 | POLK |
| ENSG00000154027 | 1 | 77747736 | 78025651 | 283 | 36 | 499492 | 4.8225 | 7.0894e-07 | AK5 |
| ENSG00000153495 | 13 | 111968531 | 111996596 | 29 | 8 | 499492 | 4.8155 | 7.343e-07 | TEX29 |
| ENSG00000135472 | 12 | 50260679 | 50298000 | 20 | 6 | 499492 | 4.8015 | 7.8731e-07 | FAIM2 |
| ENSG00000113163 | 5 | 74664311 | 74807963 | 29 | 5 | 499492 | 4.7483 | 1.0259e-06 | COL4A3BP |
| ENSG00000123444 | 11 | 47593749 | 47600567 | 1 | 1 | 499492 | 4.7392 | 1.0726e-06 | KBTBD4 |
| ENSG00000213619 | 11 | 47586888 | 47606114 | 1 | 1 | 499492 | 4.7392 | 1.0726e-06 | NDUFS3 |
| ENSG00000152253 | 2 | 169690642 | 169769881 | 71 | 8 | 499492 | 4.7355 | 1.0926e-06 | SPC25 |
| ENSG00000213996 | 19 | 19375173 | 19384200 | 2 | 1 | 499492 | 4.7313 | 1.1155e-06 | TM6SF2 |
| ENSG00000150672 | 11 | 83166055 | 85338966 | 1631 | 113 | 499492 | 4.721 | 1.1736e-06 | DLG2 |
| ENSG00000133958 | 14 | 93799565 | 94174222 | 170 | 12 | 499492 | 4.7195 | 1.182e-06 | UNC79 |
| ENSG00000155093 | 7 | 157331750 | 158380480 | 526 | 90 | 499492 | 4.7163 | 1.2009e-06 | PTPRN2 |
| ENSG00000185352 | 13 | 96743093 | 97485671 | 439 | 33 | 499492 | 4.7148 | 1.2096e-06 | HS6ST3 |
| ENSG00000164078 | 3 | 49924435 | 49941299 | 6 | 2 | 499492 | 4.7118 | 1.2276e-06 | MST1R |
| ENSG00000165916 | 11 | 47440320 | 47447993 | 6 | 2 | 499492 | 4.7039 | 1.2759e-06 | PSMC3 |
| ENSG00000134574 | 11 | 47236493 | 47260767 | 8 | 4 | 499492 | 4.6616 | 1.5687e-06 | DDB2 |
| ENSG00000066032 | 2 | 79412357 | 80875905 | 1678 | 169 | 499492 | 4.6572 | 1.603e-06 | CTNNA2 |
| ENSG00000091844 | 6 | 153325594 | 153452384 | 136 | 12 | 499492 | 4.6564 | 1.6086e-06 | RGS17 |
| ENSG00000109920 | 11 | 47738072 | 47788995 | 14 | 2 | 499492 | 4.6535 | 1.632e-06 | FNBP4 |
| ENSG00000069667 | 15 | 60780483 | 61521518 | 733 | 118 | 499492 | 4.6404 | 1.7385e-06 | RORA |
| ENSG00000184349 | 5 | 106712590 | 107006596 | 269 | 43 | 499492 | 4.6328 | 1.8042e-06 | EFNA5 |
| ENSG00000083067 | 9 | 73143979 | 74061820 | 863 | 67 | 499492 | 4.6165 | 1.9515e-06 | TRPM3 |
| ENSG00000166436 | 11 | 8633584 | 8693413 | 33 | 3 | 499492 | 4.6154 | 1.9614e-06 | TRIM66 |
| ENSG00000165923 | 11 | 47681143 | 47736941 | 16 | 3 | 499492 | 4.6031 | 2.081e-06 | AGBL2 |
| ENSG00000186094 | 1 | 48998527 | 50489585 | 619 | 31 | 499492 | 4.5986 | 2.127e-06 | AGBL4 |
| ENSG00000136944 | 9 | 129376722 | 129463311 | 69 | 15 | 499492 | 4.588 | 2.2372e-06 | LMX1B |
| ENSG00000030066 | 11 | 47799639 | 47870107 | 25 | 3 | 499492 | 4.5866 | 2.2531e-06 | NUP160 |
| ENSG00000187391 | 7 | 77646393 | 79082890 | 1560 | 159 | 499492 | 4.538 | 2.84e-06 | MAGI2 |
| ENSG00000066382 | 11 | 30406040 | 30608419 | 212 | 22 | 499492 | 4.5365 | 2.8596e-06 | MPPED2 |
| ENSG00000145934 | 5 | 166711804 | 167691162 | 727 | 97 | 499492 | 4.5356 | 2.8717e-06 | TENM2 |
| ENSG00000104447 | 8 | 116420724 | 116821899 | 234 | 12 | 499492 | 4.5309 | 2.9367e-06 | TRPS1 |


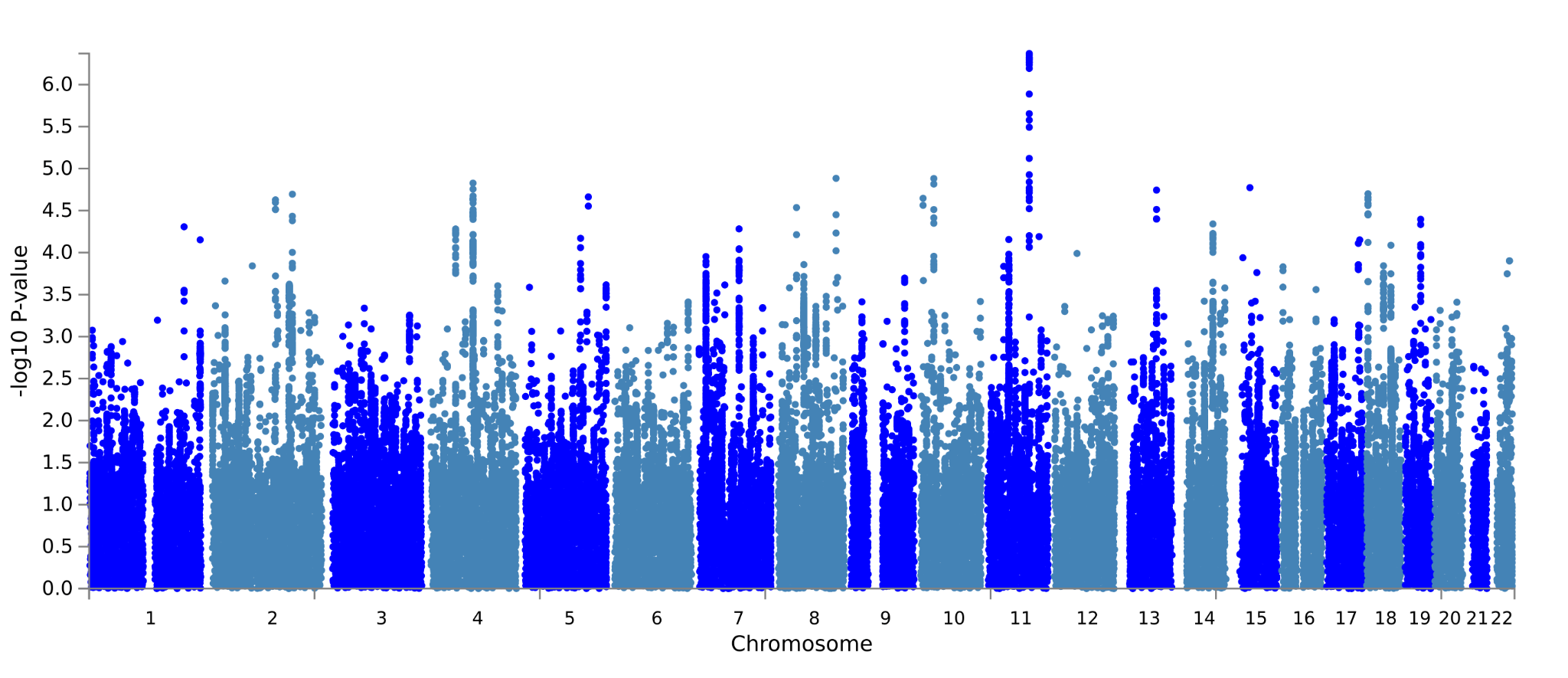


Fig. S3: Manhattan plot of GWAS summary statistics of OCD/OCS-IR latent factor. The x-axis displays the chromosomes, and the y-axis displays the −log p-value. The genome-wide significance threshold (p=5e-8).


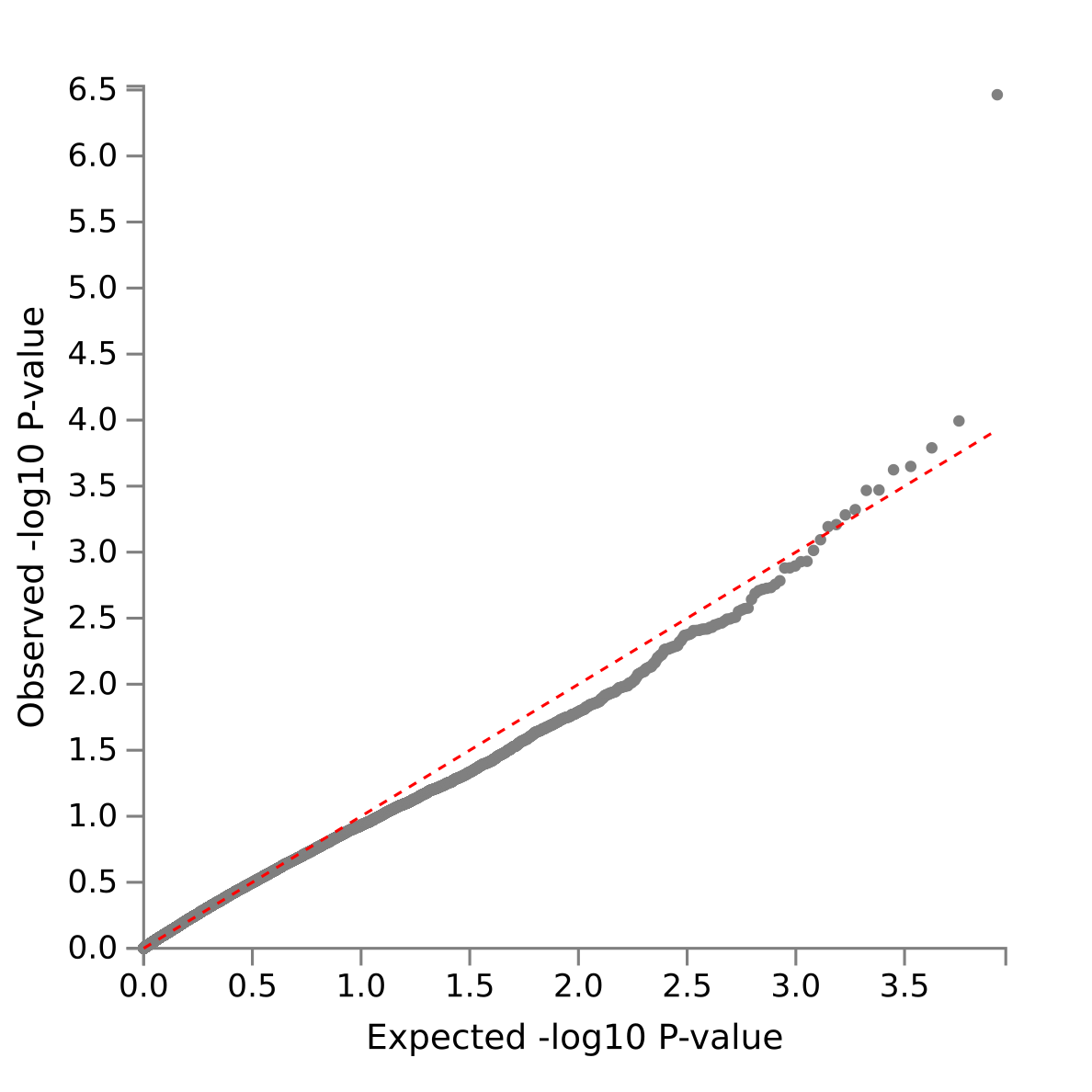


Figure S5:. Q-Q plots of OCD/OCS-IR latent factor. Quantile-quantile (Q-Q) plot of GWAS summary statistics of OCD/OCS-IR latent factor. The x-axis displays the expected −log p-value, and the y-axis displays the observed p-value.

Table S3. Top 10 SNPs of GWAS summary statistics of OCD/OCS-IR latent factor. Top 10 significant SNPs of the input GWAS summary statistics of the OCD/OCS-IR latent factor after removal of significant Q SNPs. Genome wide significance threshold was set at p=5e−8. chr=chromosome; bp=base pair position; non_effect_allele=non-effect allele ; effect_allele=effect allele ; rsID=variant ID; p=p-value; beta=beta value; se=standard error.


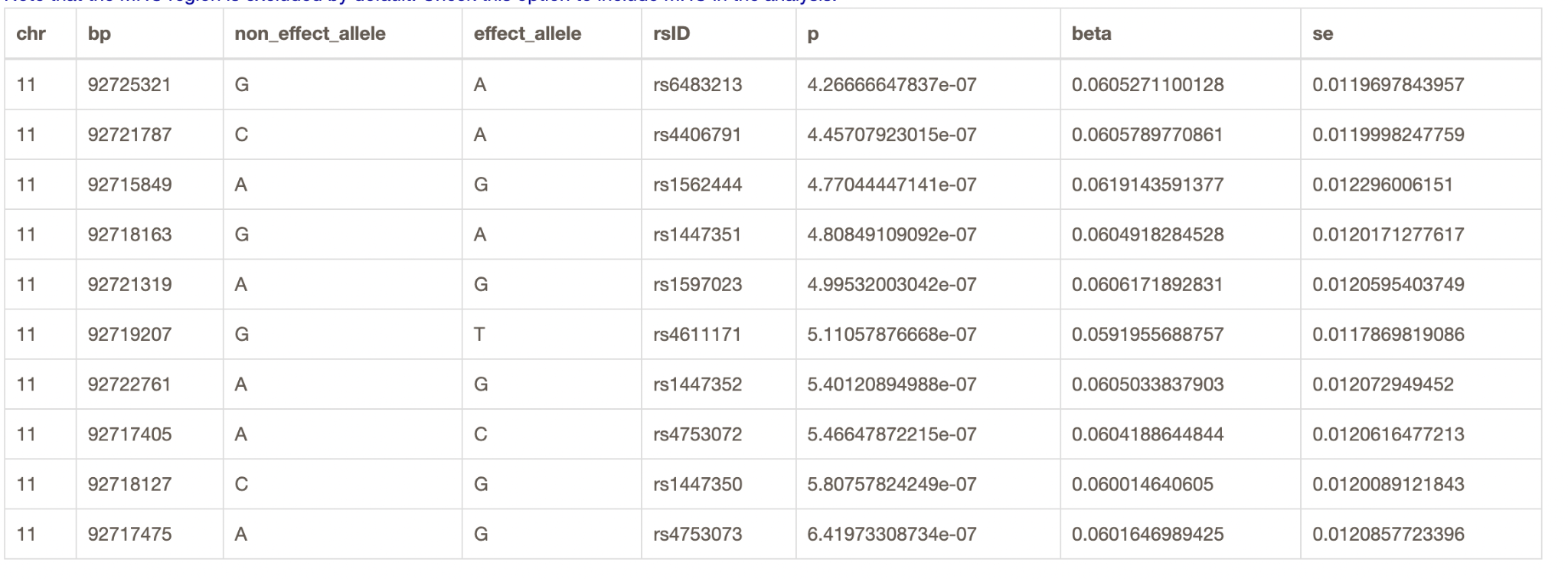


Table 2: Results of the MAGMA gene set based analysis of ADHD-IR latent factor. The table shows the top significant identified gene sets based on Bonferroni corrected p-values accounting for 5917 Gene Ontology (GO) terms and 4761 curated gene sets. N genes= number of genes; Beta=beta value; Beta STD=beta value standard deviation; SE=standard error; P=p-value ; Pbon=p-value after Bonferroni correction.

| Gene set | N genes | Beta | Beta STD | SE | P | Pbon |
| --- | --- | --- | --- | --- | --- | --- |
| GO_bp:go_neuron_differentiation | 1162 | 0.16823 | 0.042594 | 0.029012 | 3.4126e-09 | 5.28202228e-05 |
| GO_bp:go_neuron_development | 956 | 0.16921 | 0.039113 | 0.031859 | 5.5211e-08 | 0.000854500647 |
| GO_bp:go_neurogenesis | 1383 | 0.14199 | 0.038943 | 0.02681 | 5.9978e-08 | 0.000928219528 |
| GO_bp:go_positive_regulation_of_biosynthetic_process | 1692 | 0.1206 | 0.036219 | 0.02396 | 2.4373e-07 | 0.00377172175 |
| GO_bp:go_negative_regulation_of_signaling | 1159 | 0.13615 | 0.034429 | 0.028454 | 8.6411e-07 | 0.01337123814 |
| GO_bp:go_positive_regulation_of_gene_expression | 1669 | 0.11499 | 0.034324 | 0.024369 | 1.1983e-06 | 0.0185412959 |
| GO_mf:go_dna_binding_transcription_factor_activity | 1443 | 0.13257 | 0.037068 | 0.028133 | 1.2356e-06 | 0.0191172032 |
| GO_bp:go_negative_regulation_of_biosynthetic_process | 1272 | 0.12736 | 0.03362 | 0.027117 | 1.3335e-06 | 0.0206305785 |
| GO_bp:go_cellular_response_to_endogenous_stimulus | 1183 | 0.13286 | 0.033918 | 0.028819 | 2.0294e-06 | 0.031394818 |
| GO_bp:go_regulation_of_nervous_system_development | 790 | 0.16366 | 0.034567 | 0.035686 | 2.2771e-06 | 0.0352244599 |
| GO_bp:go_signal_release | 408 | 0.22339 | 0.034309 | 0.048843 | 2.4156e-06 | 0.0373645008 |
| GO_bp:go_positive_regulation_of_rna_biosynthetic_process | 1378 | 0.12058 | 0.033017 | 0.026425 | 2.5407e-06 | 0.0392970069 |

Table 3: Results of the MAGMA gene set based analysis of OCD/OCS-IR latent factor. The table shows the top significant identified gene sets based on Bonferroni corrected p-values accounting for 5917 Gene Ontology (GO) terms and 4761 curated gene sets. N genes= number of genes; Beta=beta value; Beta STD=beta value standard deviation; SE=standard error; P=p-value ; Pbon=p-value after Bonferroni correction.

| Gene set | N genes | Beta | Beta STD | SE | P | Pbon |
| --- | --- | --- | --- | --- | --- | --- |
| GO_bp:go_positive_regulation_of_rna_biosynthetic_process | 1378 | 0.097151 | 0.026599 | 0.019947 | 5.6251e-07 | 0.00870652978 |
| GO_mf:go_dna_binding_transcription_factor_activity | 1444 | 0.10013 | 0.028003 | 0.021234 | 1.2167e-06 | 0.0188308659 |
| GO_bp:go_neuron_differentiation | 1162 | 0.10139 | 0.025669 | 0.021905 | 1.8554e-06 | 0.0287141704 |
| GO_cc:go_nucleoplasm_part | 957 | 0.10355 | 0.023944 | 0.022468 | 2.0439e-06 | 0.0316293525 |
| GO_bp:go_positive_regulation_of_gene_expression | 1669 | 0.084632 | 0.02526 | 0.018406 | 2.1496e-06 | 0.0332629104 |

Fig. S6: MAGMA tissue expression analyses of tissue specificity of the genes related to ADHD-IR latent factor. MAGMA gene-property analysis results for gene expression of the selected GTEx/v8 30 general tissue types (top;A), GTEx/v8 53 specific tissue types (middle;B) and BrainSpan (bottom;C) gene expression datasets.


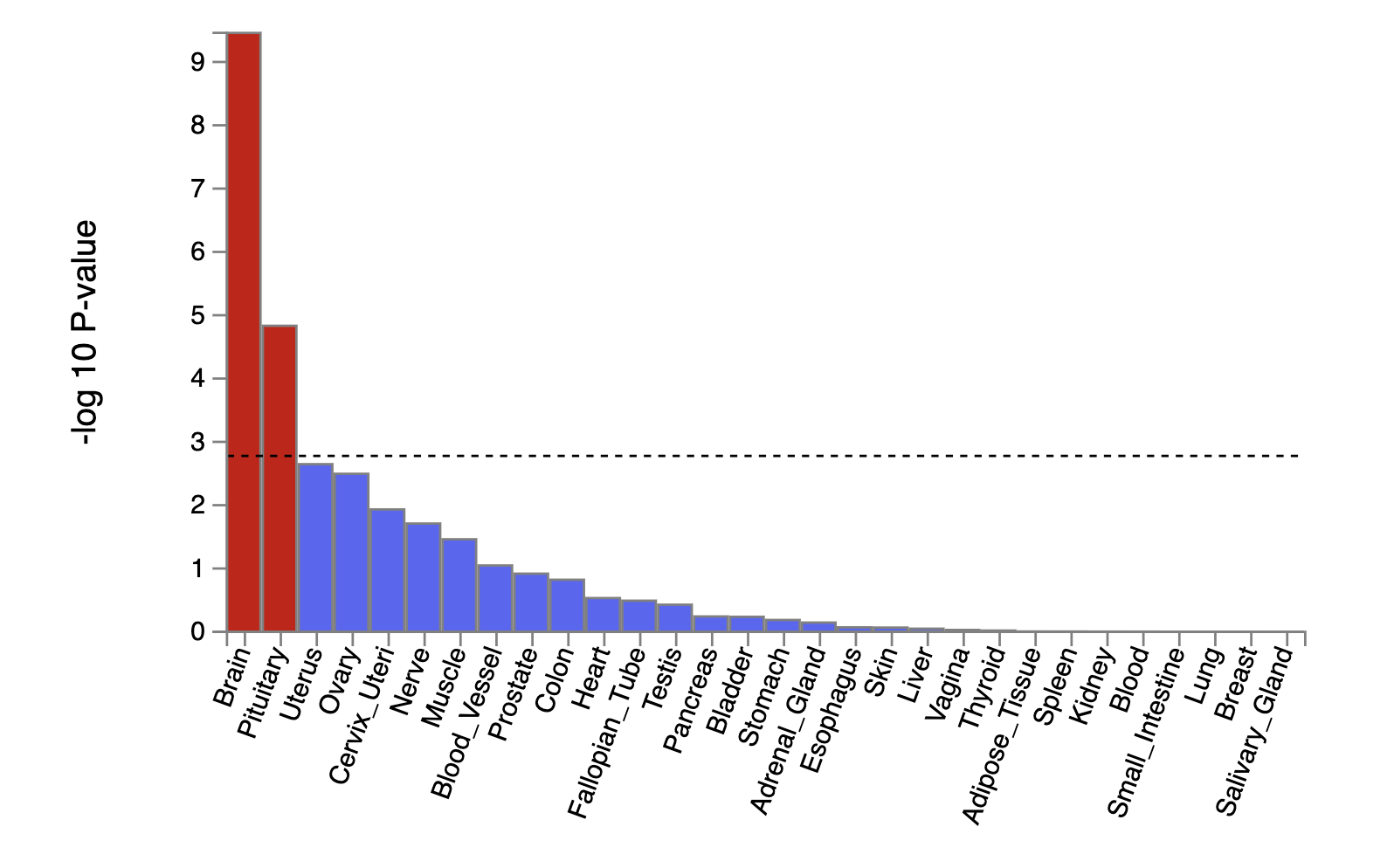


A

.


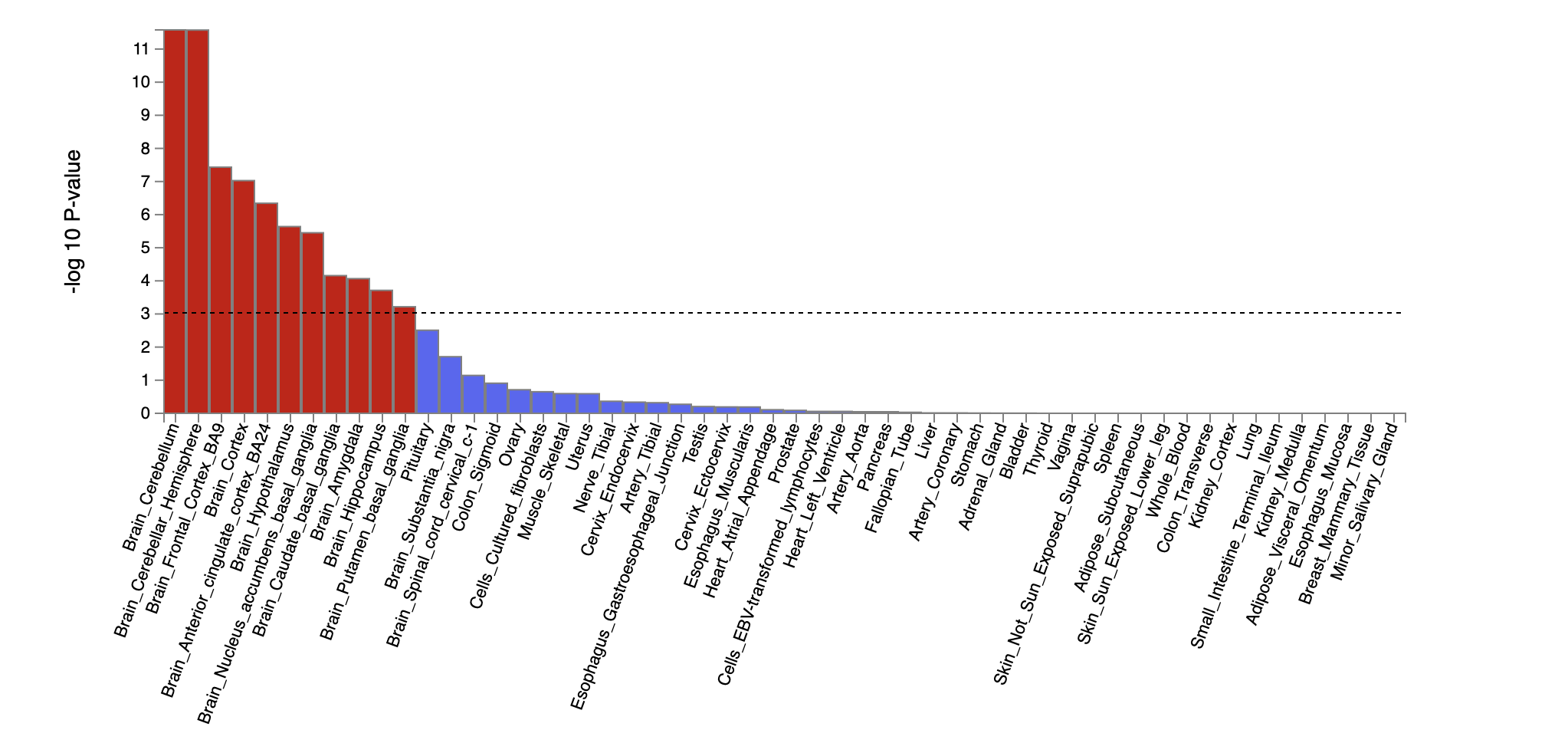


B


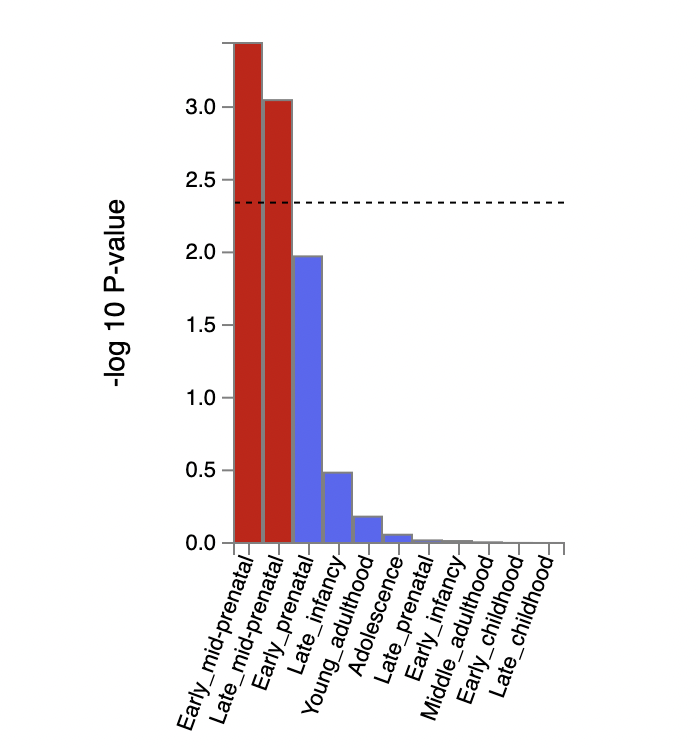


C

Fig. S7: MAGMA tissue expression analyses of tissue specificity of the genes related OCD/OCS-IR latent factor. MAGMA gene-property analysis results for gene expression of the selected GTEx/v8 30 general tissue types (top;A), GTEx/v8 53 specific tissue types (middle;B) and BrainSpan (bottom;C) gene expression datasets.


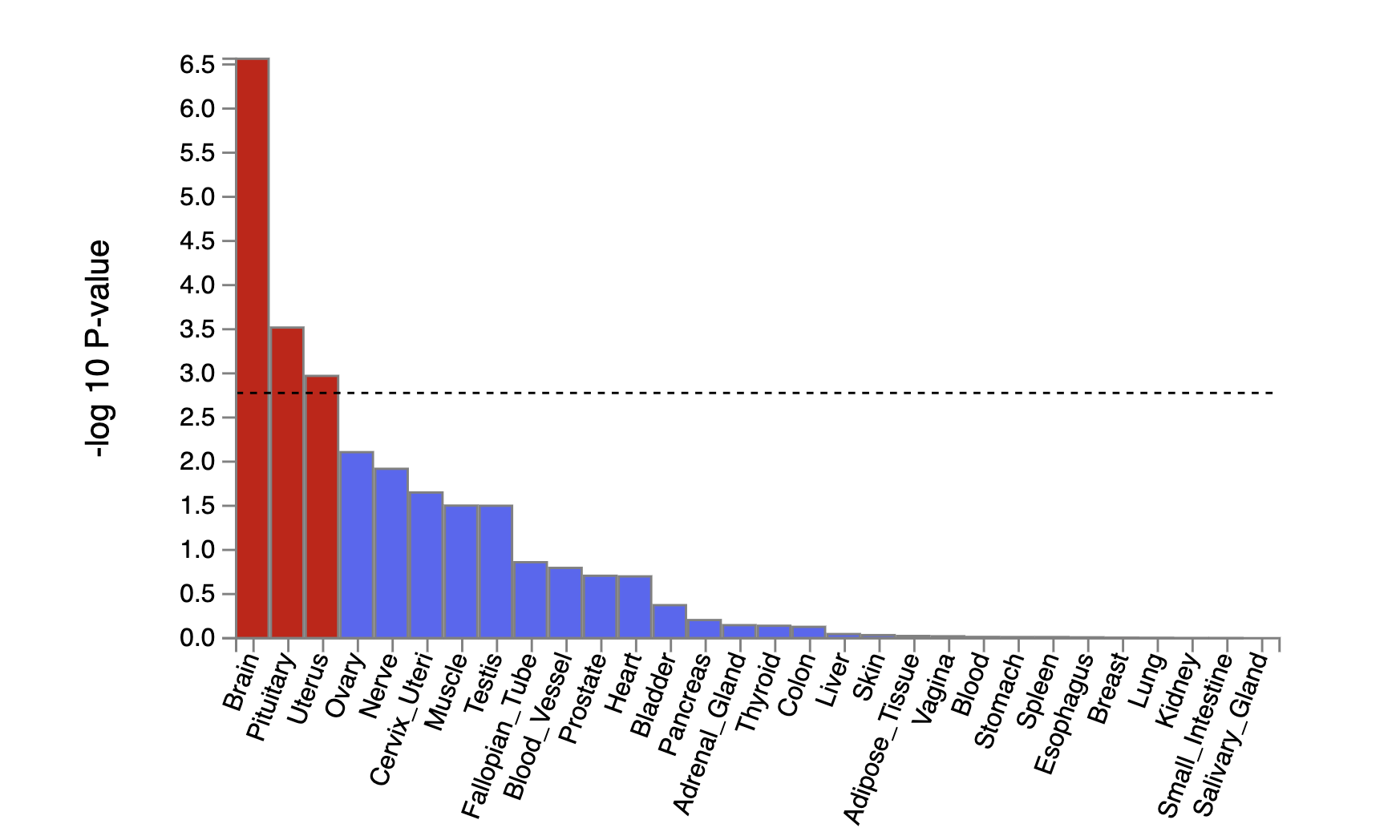


A


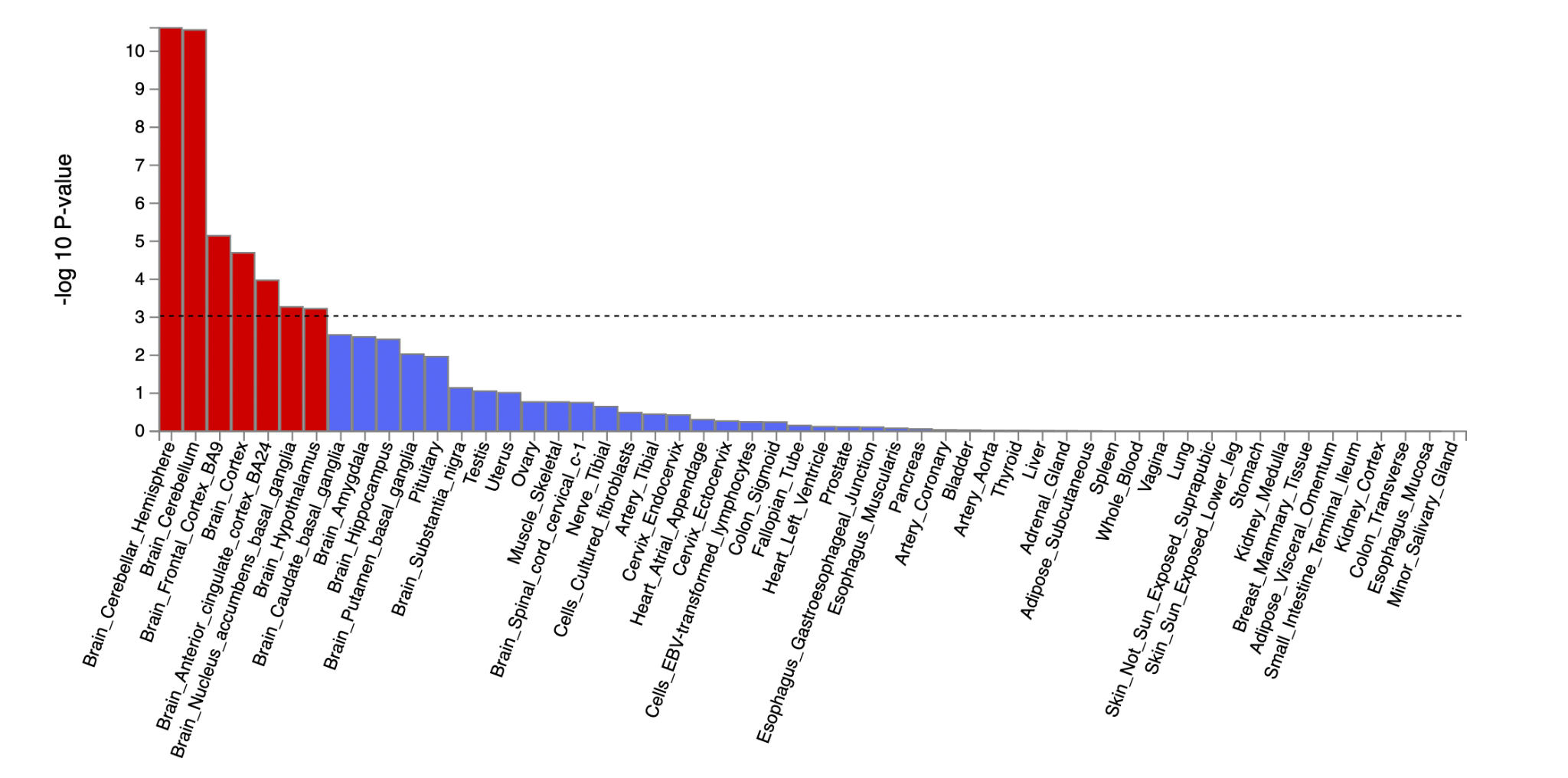


B


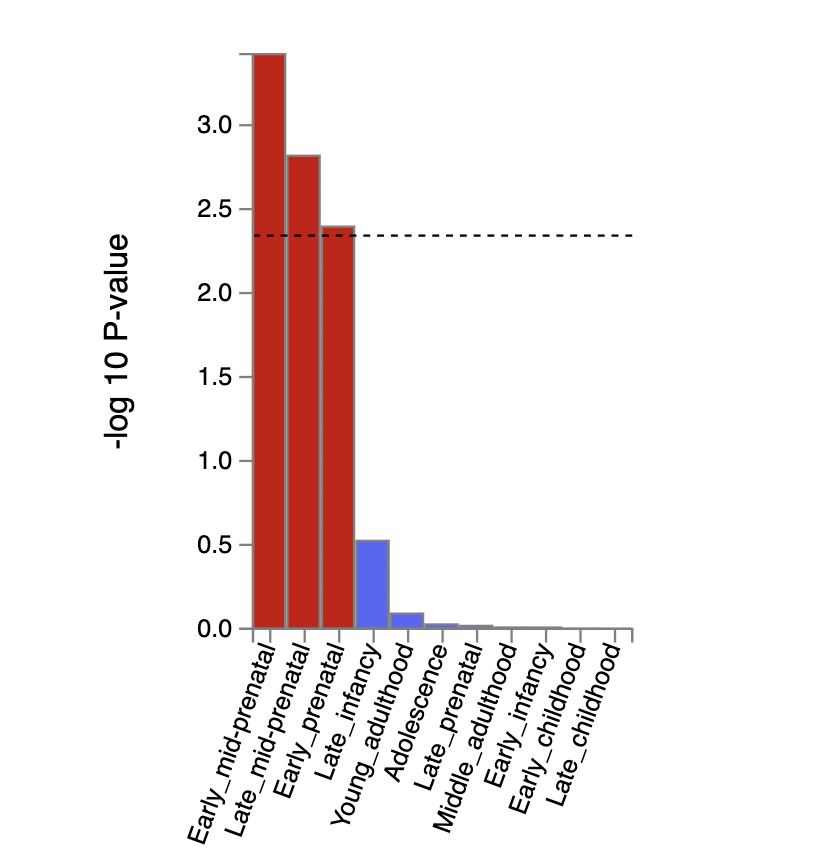


C
